# Regulation of pexophagy by a novel TBK1-MARCHF7-PXMP4-NBR1 axis in PEX1-depleted HeLa cells

**DOI:** 10.1080/15548627.2025.2593585

**Published:** 2025-11-27

**Authors:** Yong Hwan Kim, Joon Bum Kim, Ji-Eun Bae, Na Yeon Park, Seong Hyun Kim, Jae-Young Um, Dong-Seok Lee, Kyu Sun Lee, Peter K. Kim, Doo Sin Jo, Dong-Hyung Cho

**Affiliations:** a School of Life Sciences, BK21 FOUR KNU Creative BioResearch Group, Kyungpook National University, Daegu, Republic of Korea; b Organelle Institute, Kyungpook National University, Daegu, Republic of Korea; c Department of Science in Korean Medicine, Graduate School, Kyung Hee University, Seoul, Republic of Korea; d Metabolism and Neurophysiology Research Group, KRIBB, Daejeon, Republic of Korea; e Department of Biochemistry, University of Toronto, Toronto, ON, Canada; f ORGASIS Corp., Suwon, Republic of Korea

**Keywords:** MARCHF7, peroxisome, PEX1, pexophagy, PXMP4, TBK1

## Abstract

Peroxisomes are essential for lipid metabolism and redox balance, with pexophagy playing a critical role in maintaining cellular homeostasis. However, the regulatory mechanisms of pexophagy remain unclear. Through functional screening, we identified MARCHF7 as a novel E3 ligase regulating pexophagy. MARCHF7 depletion impaired pexophagic flux under PEX1 knockdown conditions. MARCHF7 binds to PXMP4 and promotes its ubiquitination at lysine 20 in PEX1-deficient cells. Depletion of PXMP4 impairs pexophagy, and reconstitution with the PXMP4 lysine 20 ubiquitination-defective mutant failed to rescue pexophagy. PEX1 depletion also induces TBK1 phosphorylation at serine 172, activating TBK1, which subsequently phosphorylates MARCHF7. This activation is driven by ROS accumulation, which reduces PXMP4 ubiquitination and prevents peroxisome loss. Furthermore, downregulation of MARCHF7 or PXMP4 impairs NBR1 recruitment to peroxisomes, suggesting that ubiquitinated PXMP4 acts as a recognition signal for NBR1. Collectively, our findings establish the TBK1-MARCHF7-PXMP4-NBR1 axis as a key regulatory pathway for pexophagy in response to PEX1 depletion.

## Introduction

Peroxisomes are essential for various cellular functions, including lipid metabolism, redox homeostasis, cellular signaling, and defense mechanism. Peroxisomes break down very-long-chain fatty acids (VLCFAs) by β-oxidation and the synthesis of plasmalogens, which are crucial for membrane integrity in many organs [1–4]. They also play a critical role in metabolic regulation by generating and eliminating reactive oxygen species (ROS) [5,6]. A primary source of peroxisomal ROS is hydrogen peroxide (H_2_O_2_), produced during VLCFA β-oxidation [7,8]. CAT (catalase) in peroxisomes maintains redox balance by scavenging these ROS [9,10]. In addition, peroxisomes serve as platforms for antiviral signaling, regulating interferon-independent defense factors to provide short-term protection against pathogens [11]. Therefore, both quantity and quality control mechanisms, including peroxisomal degradation and biogenesis, are crucial for overall cellular homeostasis [12–14].

Peroxisomal biogenesis factors, including peroxisomal biogenesis factors/peroxins (PEX) are particularly important for sustaining peroxisome function and controlling their abundance [15,16]. Peroxisomal proteins are synthesized in the cytosol and targeted to peroxisomes through peroxisomal targeting signals (PTS1, PTS2, and mPTS), which are recognized by PEX proteins [17–19]. Most peroxisomal matrix proteins contain a C-terminal PTS1 sequence [20], which is imported by the receptor PEX5 [21,22] via its tetratricopeptide repeat/TPR domain [21,23]. Other peroxisomal membrane proteins, meanwhile, use an mPTS that engages PEX19 for trafficking from the cytosol to peroxisomes [13,24]. Following this targeting step, docking with PEX3 or PEX16 facilitates peroxisome assembly [25].

Pexophagy is a selective form of macroautophagy/autophagy that eliminates surplus, damaged or dysfunctional peroxisomes to maintain peroxisomal homeostasis [14,26]. Consequently, both excessive and impaired pexophagy are associated with various pathological conditions, including peroxisomal disorders, neurodegenerative diseases, and metabolic syndromes [27–29]. Multiple stressors, such as oxidative stress, hypoxia, genetic mutations, and nutrient deprivation can induce pexophagy [26,30–33]. Notably, mutations of *PEX* genes, encoding key components involved in peroxisomal biogenesis, disrupt peroxisome maintenance and drive excessive pexophagy by increasing peroxisomal ROS production [27,32,34,35]. For example, in Zellweger syndrome, PEX1 mutations lead to peroxisomal dysfunction, resulting in peroxisome loss and severe metabolic defects [27,36]. Moreover, impaired pexophagy resulting from the dysfunction of pexophagy-related factors, such as PEX1 and HSPA9 (heat shock protein family A (Hsp70) member 9), has been implicated in the pathogenesis of various diseases, including neurodegenerative disorders, where peroxisomal stress and elevated ROS contribute to the exacerbation of cellular damage [14,29,37–39]. In addition, our group recently showed that the loss of PEX13 leads to the accumulation of ubiquitinated PEX5 on peroxisomes and an increase in peroxisome-derived ROS to trigger pexophagy [32].

Organelle selective autophagy is generally categorized into ubiquitin-dependent and ubiquitin-independent pathways [40]. In mitophagy, the PINK1 (PTEN induced kinase 1)-PRKN/Parkin pathway is the most well-known ubiquitin-dependent mechanism governing mitochondrial quality control [41]. Under stress conditions, such as mitochondrial membrane depolarization, the sensor kinase PINK1 accumulates on the outer mitochondrial membrane and phosphorylates both ubiquitin and the E3 ubiquitin ligase PRKN, leading to its activation [42]. Activated E3 PRKN ubiquitinates several specific mitochondrial membrane proteins, including VDAC1 (voltage dependent anion channel 1) and MFN2 (mitofusin 2), marking them for dysfunctional mitochondria [43–46]. These ubiquitinated substrates then serve as recognition signals for autophagy receptor proteins, such as SQSTM1/p62, which facilitate the recruitment of the autophagic machinery, ultimately driving mitophagy [43,47]. Similarly, endoplasmic reticulum (ER) selective autophagy (ERphagy) is essential for ER homeostasis [48–50]. PINK1 also regulates another E3 ligase protein, KEAP1 (kelch like ECH associated protein 1), promoting the ubiquitination of ER membrane protein, RTN1 (reticulon 1) and facilitating ERphagy in a ubiquitin-dependent manner [50]. Additionally, our group recently identified the E3 ubiquitin ligase ITCH (itchy E3 ubiquitin protein ligase) and the melanosome membrane protein MLANA (melan-A) as key components of a regulatory axis that governs melanophagy, a selective autophagy pathway responsible for the degradation of melanosomes [51,52]. Similar to other forms of organelle-selective autophagy, E3 ubiquitin ligases have been reported to ubiquitinate peroxisomal proteins, promoting the recruitment of receptor proteins that mediate pexophagy [53,54]. For instance, under nutrient deprivation, the E3 ubiquitin ligase MARCHF5 (membrane associated ring-CH-type finger 5) is recruited to peroxisomes, where it ubiquitinates the peroxisomal membrane protein ABCD3 (ATP binding cassette subfamily D member 3), promoting pexophagy [53]. Additionally, the PEX2-PEX10-PEX12 E3 ubiquitin ligase complex ubiquitinates ABCD3 and PEX5 in response to oxidative stress or nutrient deprivation, further facilitating peroxisomal degradation [30,33]. Under oxidative stress conditions, PEX5 is phosphorylated at serine 141 by ATM (ATM serine/threonine kinase) kinase, enhancing its ubiquitination by the PEX2-PEX10-PEX12 complex and triggering pexophagy [30]. In contrast, the deubiquitinating enzyme, such as USP30 (ubiquitin specific peptidase 30) counteracts the activity of the PEX2-PEX10-PEX12 complex by removing ubiquitin from peroxisomal proteins, thereby negatively regulating pexophagy [55].

Although several regulatory pathways in selective autophagy have been identified, the precise connection between sensor kinases, E3 ubiquitin ligases, ubiquitination targets, and receptor proteins in pexophagy remains unclear. In this study, we used PEX1 depletion, a well-established trigger for pexophagy, to identify for novel regulators. Our findings reveal that the TBK1-MARCHF7-PXMP4 axis constitutes a key ubiquitin-mediated pathway controlling pexophagy. These findings help our understanding of peroxisomal quality control and provide insights into potential therapeutic approaches for peroxisome-related diseases.

## Results

### E3 ubiquitin ligase MARCHF7 mediates PEX1 depletion-induced pexophagy

**Figure 1.**
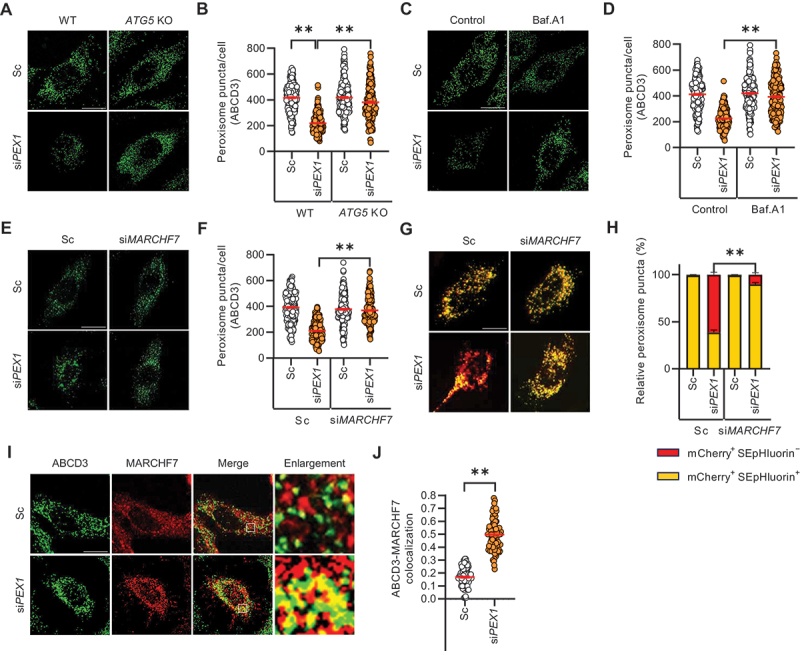
E3 ubiquitin ligase MARCHF7 mediates PEX1 depletion-induced pexophagy. (**A and B**) wild-type (WT) and *ATG5*-knockout (KO) HeLa cells were transiently transfected with either scrambled siRNA (SC) or validated siRNA targeting *PEX1* (si*PEX1*) for 72 h. Cells were then immunostained with an anti-ABCD3 antibody. The number of peroxisomes per cell was quantified (*n* ≥200). (**C and D**) HeLa cells were transiently transfected with Sc or si*PEX1* for 24 h, followed by treatment with bafilomycin A_1_ (Baf.A1, 10 nM) for an additional 48 h. Cells were then stained with an anti-ABCD3 antibody. The number of peroxisomes per cell was quantified (*n* ≥200). (**E and F**) HeLa cells were transiently transfected with Sc, si*PEX1*, or siRNA targeting *MARCHF7* (si*MARCHF7*) for 72 h. Cells were then fixed, immunostained with an anti-ABCD3 antibody, and imaged using confocal microscopy. The number of peroxisomes per cell was quantified (*n* ≥200). (**G and H**) HeLa cells were co-transfected with mCherry-SEpHluorin-SKL and either si*PEX1* or si*MARCHF7* for 72 h. Cells were imaged using fluorescence microscopy, and the proportion of mCherry^+^ SEpHluorin^+^ peroxisomes and mCherry^+^ SEpHluorin^−^ pexolysosomes was calculated (*n* ≥100). (**I and J**) HeLa cells were transfected with Sc or si*PEX1* for 72 h. Cells were then fixed, immunostained with anti-ABCD3 and anti-MARCHF7 antibodies, and imaged using confocal microscopy. Pearson’s correlation coefficient was used to quantify the colocalization between ABCD3 and MARCHF7. The graph represents the colocalization analysis results obtained using the Coloc2 plugin in Fiji, based on Pearson’s correlation coefficient (*n* ≥100). Scale bar: 20 µm. Data are presented as mean ± standard error of the mean (SEM); **p < 0.001.

Previously, we and other group have demonstrated that PEX1 is involved in the import of matrix proteins into peroxisomes and is implicated in pexophagy regulation [34,56,57]. Given that PEX1 mutations are frequently associated with a reduction in peroxisome numbers [58,59], we hypothesized that multiple regulatory factors are involved in pexophagy in response to PEX1 depletion. To validate this hypothesis, we transiently transfected both wild-type (WT) and *ATG5* (autophagy related 5) knockout (KO) HeLa cells with scrambled siRNA or *PEX1*-targeting siRNA (si*PEX1*). Immunostaining for peroxisomes with ABCD3 protein revealed a significant loss of peroxisomes in WT cells upon PEX1 depletion, whereas this loss was completely blocked in *ATG5*-KO cells (Figure 1A,B). Additionally, treatment with bafilomycin A1 (Baf.A1), an autophagosome-lysosome fusion inhibitor, also effectively prevented the loss of peroxisome induced by depletion of PEX1 (Figure 1C,D). Furthermore, colocalization of ABCD3 with LAMP1 (lysosomal associated membrane protein 1) was significantly increased in PEX1-depleted cells (Figure S1A,B). Since impaired peroxisome biogenesis results in an overall reduction of peroxisomes, we next investigated the effect of PEX1 depletion on peroxisome biogenesis. The PPAR isotypes (A/α, D/δ, and G/γ) exhibited no significant alterations in PEX1-depleted conditions (Figure S1C), and these results implicate that PEX1 depletion-induced peroxisome loss is predominantly mediated through the autophagic machinery.

Various E3 ubiquitin ligases play a pivotal role in maintaining organelle homeostasis by mediating selective autophagy [33,43]. Previously it was reported that MARCHF5 regulates mitophagy by ubiquitination of mitophagy regulator, FUNDC1 [60]. In addition, MARCHF6 involves in the regulation of ERphagy [61]. Therefore, we synthesized and performed siRNA screen using a small-scale library of 11 MARCHF family E3 ubiquitin ligases. From the screening, we identified MARCHF7 as a potent and previously unrecognized regulator of pexophagy (Figure S2). To evaluate the role of MARCHF7 in pexophagy, HeLa cells were transiently transfected with scrambled siRNA, si*PEX1*, or si*MARCHF7*, followed by immunostaining for ABCD3. Notably, the loss of peroxisomes observed upon PEX1 depletion was further completely blocked by knockdown of MARCHF7 (Figure 1E,F). To further determine whether MARCHF7 modulates pexophagy flux, we employed a novel pexophagy monitoring method utilizing double-fluorescent protein system with mCherry-SEpHluorin fused to the peroxisomal targeting signal (PTS1). As shown in Figure 1G,H, the number of acidic compartments exhibiting red-only puncta (mCherry^+^ SEpHluorin^−^) pexolysosomes was significantly increased in PEX1-depleted cells, indicating enhanced peroxisome degradation. In contrast, peroxisomes remained visible as yellow puncta (mCherry^+^ SEpHluorin^+^) in MARCHF7-depleted cells, suggesting that pexophagy was blocked due to the loss of MARCHF7 function (Figure 1G,H). To examine MARCHF7 localization under PEX1 depletion, we assessed MARCHF7 colocalization with ABCD3. Notably, MARCHF7 exhibited increased colocalization with peroxisomes in PEX1-depleted cells (Figure 1I,J). Collectively, these findings suggest that MARCHF7 plays an essential role in mediating PEX1 depletion-induced pexophagy.

### MARCHF7 mediates PXMP4 ubiquitination dependent pexophagy in PEX1-depleted cells

**Figure 2.**
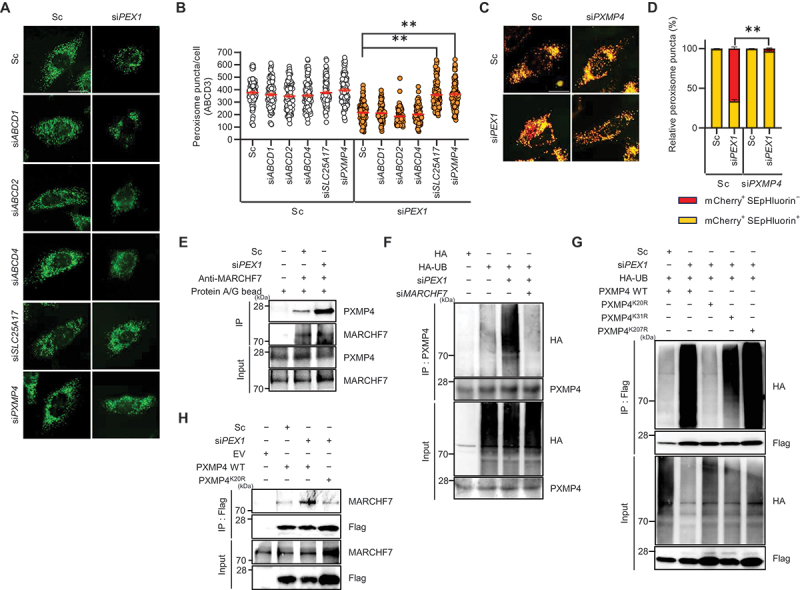
MARCHF7 mediates PXMP4 (lysine 20) ubiquitination dependent pexophagy in PEX1-depleted cells. (**A and B**) HeLa cells were transiently transfected with Sc or si*PEX1* in combination with siRnas targeting peroxisomal membrane proteins for 72 h. Cells were then immunostained with an anti-ABCD3 antibody, and the number of peroxisomes per cell was quantified (*n* ≥100). Scale bar: 20 µm. (**C and D**) HeLa cells were co-transfected with mCherry-SEpHluorin-SKL and either si*PEX1* or si*PXMP4* for 72 h. Cells were imaged using fluorescence microscopy, and the proportion of mCherry^+^ SEpHluorin^+^ puncta and mCherry^+^ SEpHluorin^−^ puncta was calculated (*n* ≥100). (**E**) HeLa cells were transfected with Sc or si*PEX1* for 72 h, harvested, and subjected to immunoprecipitation using anti-MARCHF7 antibodies conjugated to agarose beads. Samples were analyzed by western blotting with the indicated antibodies. (**F**) HeLa cells were transfected with si*PEX1* or si*MARCHF7* along with HA and HA-ubiquitin (HA-UB) for 48 h, followed by treatment with MG132 (10 µM) for 12 h. Cells were harvested and subjected to immunoprecipitation using anti-PXMP4 antibodies conjugated to agarose beads, and samples were analyzed by western blotting with the indicated antibodies. (**G**) HeLa cells were transfected with Sc, si*PEX1*, Flag-tagged PXMP4 K/R mutants (PXMP4 wt, PXMP4^K20R^, PXMP4^K31R^, or PXMP4^K207R^) in combination with HA-UB for 48 h, followed by MG132 treatment (10 µM) for 12 h. Cells were then harvested and subjected to immunoprecipitation using anti-Flag antibodies conjugated to agarose beads, and samples were analyzed by western blotting with the indicated antibodies. (**H**) HeLa cells were transiently transfected with Sc or si*PEX1* in combinations with Flag (empty vector, ev), PXMP4 wt or PXMP4^K20R^ mutant for 48 h. After, the cells were harvested and subjected to immunoprecipitation by using agarose-conjugated anti-Flag antibody. The samples were analyzed by western blotting with the indicated antibodies. Scale bar: 20 µm. Data are presented as mean ± SEM; **p < 0.001.

Because ubiquitination of organelle membrane proteins is a crucial regulatory mechanism in organelle selective autophagy, we tried to identify peroxisomal protein targets of MARCHF7. Using a deep-learning-based webserver for protein post-translational modification site prediction (MultisiteDeep), we computationally predicted five peroxisomal membrane proteins with high ubiquitination confidence, including ABCD1, ABCD2, ABCD4, SLC25A17/PMP34 (solute carrier family 25 member 17), and PXMP4 [62]. Then we further examined by a subsequent siRNA screening under PEX1 depletion conditions and selected PXMP4 (peroxisomal membrane protein 4) and SLC25A17 as key regulators of peroxisome loss (Figure 2A,B). Additionally, the number of acidic compartments containing red-only puncta (mCherry^+^ SEpHluorin^−^) was markedly increased in PEX1-depleted cells. In contrast, yellow puncta (mCherry^+^ SEpHluorin^+^) were predominantly exhibited in PXMP4-depleted cells, indicating that pexophagy was inhibited by the loss of the ubiquitination substrate PXMP4 (Figure 2C,D). Given the strong functional link to pexophagy, we focused on PXMP4 as a candidate substrate of MARCHF7. Immunoprecipitation assays showed direct interaction between MARCHF7 and PXMP4, with this interaction being significantly enhanced upon PEX1 depletion (Figure 2E and S3D). Furthermore, we observed colocalization of MARCHF7 with PXMP4 under PEX1-depleted cells (Figure S3G,H).

To further explore whether PXMP4 is a ubiquitination target for MARCHF7, we performed ubiquitination assays in cells transfected with si*PEX1* and si*MARCHF7*. Notably, the results revealed that PEX1 depletion markedly increased PXMP4 ubiquitination, whereas this effect was abolished by MARCHF7 knockdown (Figure 2F). Further computational prediction using MultisiteDeep identified eight putative ubiquitination sites on PXMP4 (K20, K31, K42, K63, K86, K98, K145, K207). Topology analysis using SPOCTOPUS software indicated that K20, K31, and K207 are located on the cytosolic face, implicating them likely ubiquitination sites [63]. Then, we examined the functional relevance of PXMP4 by generating mutants in which lysine residues (K20, K31, or K207) were substituted with arginine (R), preventing ubiquitination at these sites. Remarkably, ubiquitination assays revealed that only the K20R mutant highly abolished the increased ubiquitination of PXMP4 in PEX1-depleted cells, whereas K31R and K207R had no effect (Figure 2G). Furthermore, immunoprecipitation assays demonstrated that the interaction between MARCHF7 and PXMP4 WT was enhanced upon PEX1 depletion, whereas the interaction with PXMP4^K20R^ was abolished (Figure 2H). Collectively, these findings suggest that PXMP4 is a novel peroxisomal ubiquitination target for MARCHF7 in response to PEX1 depletion.

### PXMP4 lysine 20 ubiquitination is required for pexophagy in PXMP4-depleted cells

**Figure 3.**
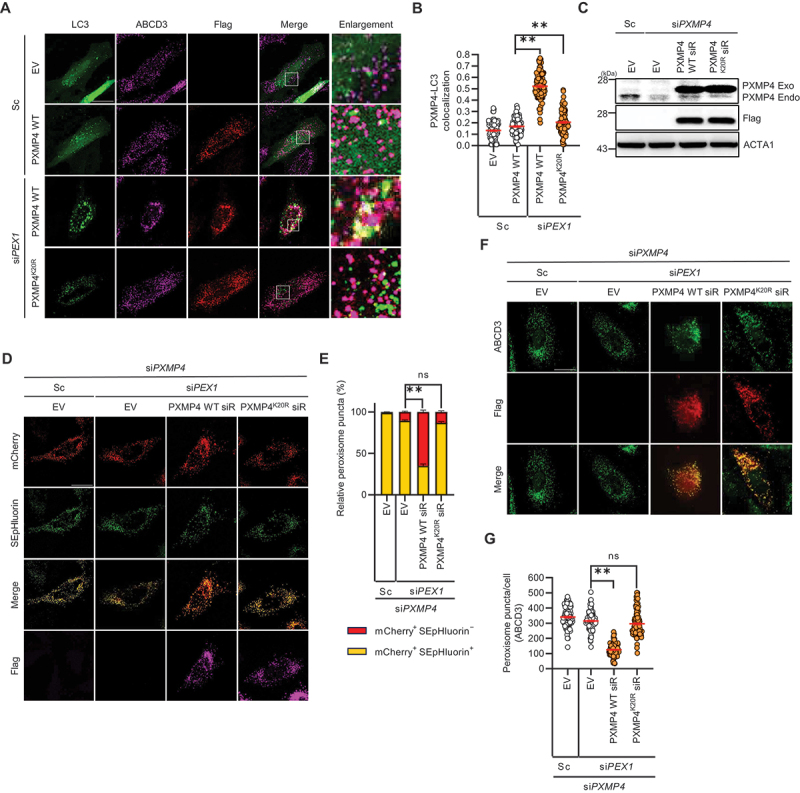
Loss of ubiquitination at PXMP4 lysine 20 disrupts pexophagy in PXMP4-depleted cells. (**A and B**) HeLa cells were co-transfected with GFP-LC3 and either PXMP4 wt or PXMP4^K20R^ vectors together with Sc or si*PEX1* for 48 h. Cells were then fixed, immunostained with anti-ABCD3 and anti-Flag antibodies, and imaged using confocal microscopy. Pearson’s correlation coefficient was used to quantify the colocalization between Flag (PXMP4) and GFP-LC3. The graph represents the colocalization analysis results obtained using the Coloc2 plugin in Fiji, based on Pearson’s correlation coefficient (*n* ≥100). (**C**) HeLa cells were transfected with Sc, si*PXMP4*, ev, Flag-tagged PXMP4 wt siRNA-resistant vector (PXMP4 wt siR), and Flag-tagged PXMP4^K20R^ siRNA-resistant vector (PXMP4^K20R^ siR) vectors for 48 h, and the samples were analyzed by western blotting with the indicated antibodies. (**D and E**) HeLa cells were transiently co-transfected with mCherry-SEpHluorin-SKL and PXMP4 wt siR or PXMP4^K20R^ siR constructs together with si*PEX1* or si*PXMP4* for 48 h. Cells were then fixed, immunostained with an anti-Flag antibody, and imaged using confocal microscopy. The proportion of mCherry^+^ SEpHluorin^+^ puncta and mCherry^+^ SEpHluorin^−^ puncta was calculated (*n* ≥100). (**F and G**) HeLa cells were transiently co-transfected with PXMP4 wt siR or PXMP4^K20R^ siR constructs together with si*PEX1* or si*PXMP4* for 48 h. Cells were immunostained with anti-ABCD3 and anti-Flag antibodies, and the number of peroxisomes per cell was quantified (*n* ≥100). Scale bar: 20 µm. Data are presented as mean ± SEM; **p < 0.001; ns, not significant.

To assess the effect of PXMP4 ubiquitination in autophagosome recruitment, we examined LC3 colocalization in PEX1-depleted cells. Notably, LC3 colocalized with peroxisomes in PXMP4 WT-overexpressing cells but not in K20R-overexpressing cells (Figure 3A,B). To further investigate the role of the PXMP4 K20 residue, a ubiquitination target in the pexophagy pathway, we generated Flag tagged-siRNA resistant PXMP4 constructs (PXMP4 WT siR and PXMP4^K20R^ siR) by site-directed mutagenesis, and both constructs were overexpressed in HeLa cells upon siRNA-mediated depletion of endogenous PXMP4 (Figure 3C). Remarkably, reconstitution of PXMP4 WT siR rescued si*PEX1*-induced peroxisome loss and restored pexophagy flux in PXMP4-depleted cells. In contrast, the PXMP4^K20R^ siR mutant failed to rescue peroxisome loss or pexophagy flux under PEX1-depleted conditions (Figure 3D–G). Taken together, these findings indicate that disruption of PXMP4 ubiquitination at K20 suppresses PEX1 depletion-induced pexophagy.

### TBK1 is activated and phosphorylates MARCHF7 in response to PEX1 depletion

**Figure 4.**
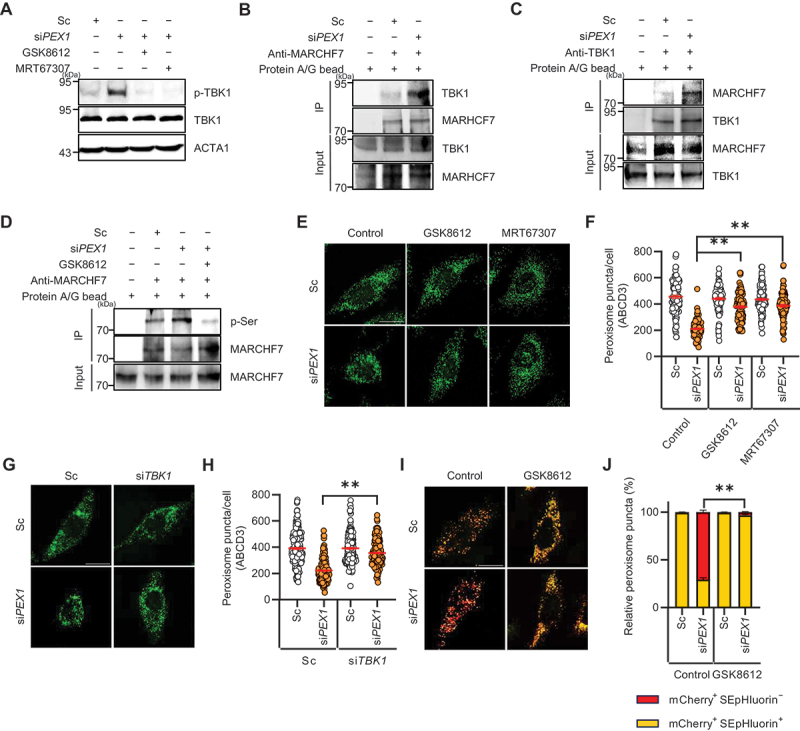
TBK1 activation and MARCHF7 phosphorylation in response to PEX1 depletion. (**A**) HeLa cells were transfected with Sc or si*PEX1* for 72 h, followed by treatment with or without GSK8612 (10 µM) or MRT67307 (2 µM) for 24 h. Cells were harvested and analyzed by western blotting with the indicated antibodies. (**B**) HeLa cells were transfected with Sc or si*PEX1* for 72 h, harvested, and subjected to immunoprecipitation using anti-MARCHF7 antibodies conjugated to agarose beads. Samples were analyzed by western blotting with the indicated antibodies. (**C**) HeLa cells were transfected with Sc or si*PEX1* for 72 h, harvested, and subjected to immunoprecipitation using anti-TBK1 antibodies conjugated to agarose beads. Samples were analyzed by western blotting with the indicated antibodies. (**D**) HeLa cells were transfected with Sc or si*PEX1* for 72 h, treated with or without GSK8612 (10 µM) for 24 h, and subjected to immunoprecipitation using anti-MARCHF7 antibodies conjugated to agarose beads. Samples were analyzed by western blotting with the indicated antibodies. (**E and F**) HeLa cells were transfected with Sc or si*PEX1* for 72 h and treated with or without GSK8612 (10 µM) or MRT67307 (2 µM) for 48 h. Cells were fixed, immunostained with an anti-ABCD3 antibody, and imaged using confocal microscopy. The number of peroxisomes per cell was quantified (*n* ≥100). (**G and H**) HeLa cells were transfected with Sc, si*PEX1*, or si*TBK1* for 72 h, fixed, immunostained with an anti-ABCD3 antibody, and imaged using confocal microscopy. The number of peroxisomes per cell was quantified (*n* ≥200). (**I and J**) HeLa cells were co-transfected with mCherry-SEpHluorin-SKL and either Sc or si*PEX1* for 24 h, followed by treatment with GSK8612 (10 µM) for an additional 48 h. Cells were imaged using fluorescence microscopy, and the proportion of mCherry^+^ SEpHluorin^+^ puncta and mCherry^+^ SEpHluorin^−^ puncta was calculated (*n* ≥100). Data are presented as mean ± SEM; **p < 0.001.

Although recent studies have demonstrated that TBK1 (TANK binding kinase 1) plays a crucial role in the regulation of mitophagy and lysophagy [64,65], its involvement in pexophagy has remained largely unexplored. To investigate whether TBK1 is activated in response to PEX1 depletion, PEX1 depleted cells were treated with the TBK1 inhibitors GSK8612 and MRT67307. As shown in Figure 3A, we found that increased phosphorylated TBK1 at serine 172 upon PEX1 depletion, which was abolished by TBK1 inhibitors (Figure 4A), suggesting that TBK1 is phosphorylated and activated in response to PEX1 depletion. Thus, we examined whether TBK1 functions as an upstream kinase regulating MARCHF7. Immunoprecipitation assays demonstrated that TBK1 interacts with MARCHF7, with this interaction being markedly enhanced following PEX1 depletion (Figure 4B,C).

Next, we examined whether MARCHF7 is a phosphorylation target of TBK1 by detecting phosphorylated MARCHF7. Our results showed that phosphorylation of the serine residue in MARCHF7 was significantly increased in PEX1-depleted cells, whereas treatment with TBK1 inhibitors effectively suppressed this phosphorylation (Figure 4D). These findings suggest that TBK1 phosphorylates MARCHF7 upon PEX1 depletion. To additionally determine the functional significance of TBK1-mediated phosphorylation in pexophagy, we examined loss of peroxisome upon TBK1 inhibition. Remarkably, treatment with TBK1 inhibitors suppressed the peroxisome loss induced by PEX1 depletion (Figure 4E,F). Not only chemical inhibition, but genetic depletion of *TBK1* using siRNA also resulted in a similar protective effect, preventing peroxisome loss in PEX1 depleted cells (Figure 4G,H). To assess the effect of TBK1 inhibition on pexophagy flux, HeLa cells were transfected with mCherry-SEpHluorin-SKL together with scrambled siRNA or si*PEX1*. PEX1 depletion markedly increased red puncta (mCherry^+^ SEpHluorin^−^), and the increase was abolished by TBK1 inhibition, indicating that TBK1 is required for PEX1 depletion-induced pexophagy (Figure 4I,J). Taken together, these results strongly indicate that TBK1 acts as an upstream regulator of MARCHF7, mediating its phosphorylation in response to PEX1 depletion.

### TBK1 phosphorylation is induced by increased ROS following PEX1 depletion

**Figure 5.**
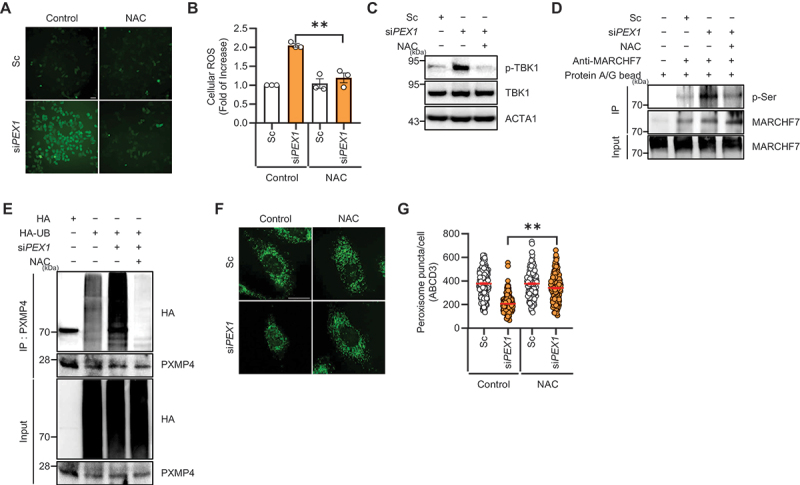
TBK1 phosphorylation is induced by ros accumulation following PEX1 depletion. (**A and B**) HeLa cells were transfected with Sc or si*PEX1* for 72 h, treated with or without nac (1 mM) for 48 h, and incubated with DCFH-DA (10 µM, 15 min). Cells were imaged, and fluorescence intensity was quantified using ImageJ. Data represent mean ± SEM from 3 independent experiments; 100 cells were analyzed per condition in each experiment. Scale bar: 50 µm. (**C**) HeLa cells were transfected with Sc or si*PEX1* for 72 h, treated with NAC (1 mM) for 48 h, and analyzed by western blotting with the indicated antibodies. (**D**) HeLa cells were transfected with Sc or si*PEX1* for 72 h, treated with NAC (1 mM) for 48 h, and subjected to immunoprecipitation using anti-MARCHF7 antibodies conjugated to agarose beads. Samples were analyzed by western blotting with the indicated antibodies. (**E**) HeLa cells were transfected with si*PEX1* along with HA and HA-UB for 48 h, then treated with MG132 (10 µM) for 12 h, with or without NAC (1 mM) for 24 h. Cells were subjected to immunoprecipitation using anti-PXMP4 antibodies conjugated to agarose beads, and samples were analyzed by western blotting. (**F and G**) HeLa cells were transfected with Sc or si*PEX1* for 72 h, treated with or without NAC (1 mM) for 48 h, fixed, immunostained with an anti-ABCD3 antibody, and imaged using confocal microscopy. The number of peroxisomes per cell was quantified (*n* ≥ 200). Scale bar: 20 µm. Data are presented as mean ± SEM; **p < 0.001.

Previous studies, including our own, have demonstrated that PEX1 depletion leads to elevated cellular ROS levels [32,35]. Given that oxidative stress can activate TBK1 in other autophagy-related pathways, we investigated whether ROS generation upon PEX1 depletion contributes to TBK1 activation. Consistently, PEX1 depletion significantly increased cellular ROS levels, an effect that was completely abolished by NAC treatment (Figure 5A,B).

Next, we examined whether ROS-mediated signaling contributes to TBK1 activation during pexophagy. Notably, PEX1 depletion-induced TBK1 phosphorylation at serine 172 was markedly suppressed upon NAC treatment, indicating that the increase in ROS upon *PEX1* depletion acts as an upstream trigger for TBK1 activation (Figure 5C). We then further examined whether ROS-mediated TBK1 activation influences MARCHF7 phosphorylation. Immunoprecipitation assays in cells transfected with si*PEX1* and treated with NAC showed that NAC treatment effectively suppressed MARCHF7 phosphorylation, suggesting that ROS-dependent TBK1 activation is responsible for MARCHF7 phosphorylation (Figure 5D). Next, we investigated the effect of ROS on PXMP4 ubiquitination and pexophagy flux. Ubiquitination assays revealed that PEX1 depletion-induced PXMP4 ubiquitination was significantly reduced upon NAC treatment (Figure 5E). Additionally, peroxisome loss induced by PEX1 depletion was completely prevented by NAC treatment (Figure 5F,G). Taken together, these results suggest that PEX1 depletion leads to increased ROS levels, which subsequently drive TBK1 activation, MARCHF7 phosphorylation, and PXMP4 ubiquitination, ultimately promoting pexophagy.

### Influence of the TBK1-MARCHF7-PXMP4-NBR1 axis on PEX1 depletion-induced pexophagy

**Figure 6.**
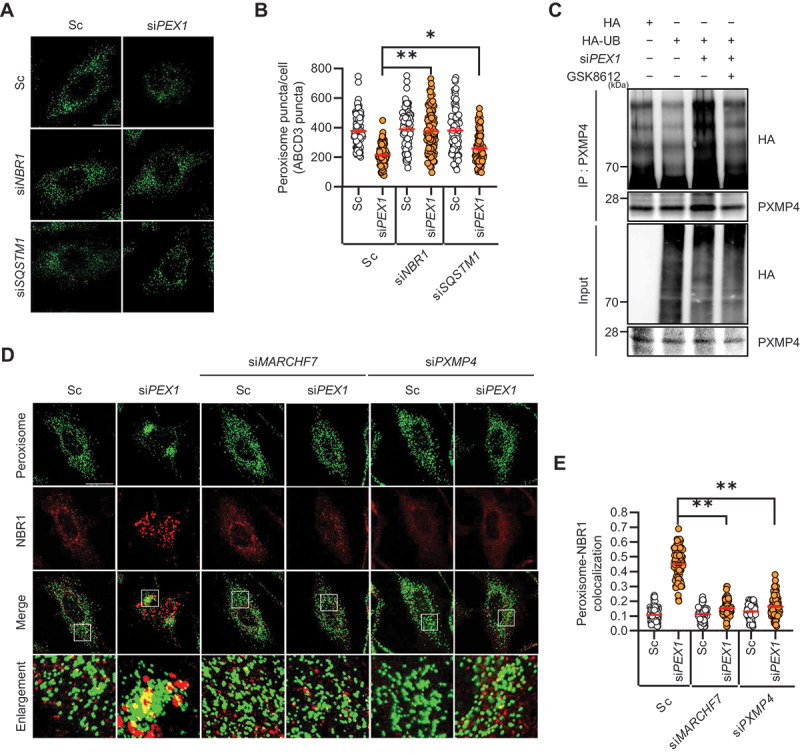
Influence of the TBK1-MARCHF7-PXMP4-NBR1 axis on pexophagy induced by PEX1 depletion. (**A and B**) HeLa cells were transiently transfected with scrambled siRNA (Sc), *PEX1* siRNA (si*PEX1*), validated *NBR1* siRNA (si*NBR1*), or validated *SQSTM1* siRNA (si*SQSTM1*) for 72 h. Cells were fixed, immunostained with an anti-ABCD3 antibody, and imaged using confocal microscopy. The number of peroxisomes per cell was quantified (*n* ≥100). (**C**) HeLa cells were transiently transfected with si*PEX1* along with HA and HA-UB for 48 h, followed by treatment with MG132 (10 µM) for 12 h, with or without GSK8612 (10 µM) for 24 h. Cells were harvested and subjected to immunoprecipitation using anti-PXMP4 antibodies conjugated to agarose beads. Samples were analyzed by western blotting with the indicated antibodies. (**D and E**) HeLa cells were transiently transfected with Sc, si*PEX1*, si*MARCHF7*, or si*PXMP4* for 72 h, then fixed and immunostained with anti-ABCD3 (peroxisome) and anti-NBR1 antibodies. Cells were imaged using confocal microscopy. Pearson’s correlation coefficient was used to quantify the colocalization between peroxisomes and NBR1. The graph represents the colocalization analysis results obtained using the coloc 2 plugin in Fiji, based on Pearson’s correlation coefficient (*n* ≥100). Scale bar: 20 µm. Data are presented as mean ± standard error of the mean (SEM); *p < 0.01, **p < 0.001.

Both NBR1 and SQSTM1 are well-characterized autophagy receptor proteins involved in pexophagy. To investigate their roles in PEX1 depletion-induced pexophagy, we assessed peroxisome loss following NBR1 or SQSTM1 knockdown. Although the depletion of either NBR1 or SQSTM1 significantly reduced peroxisome loss induced by PEX1 depletion, NBR1 exhibited a slightly stronger effect (Figure 6A,B).

Additionally, we performed ubiquitination assays to determine whether the upstream kinase TBK1 regulates PXMP4 ubiquitination. Notably, the results showed that TBK1 inhibition significantly reduced PXMP4 ubiquitination in PEX1-depleted cells (Figure 6C).

Given our findings that PEX1 depletion leads to TBK1 activation and MARCHF7-mediated PXMP4 ubiquitination, we hypothesized that NBR1 preferentially interacts with ubiquitinated PXMP4 to facilitate pexophagy. To test this, we examined whether NBR1 recruitment to peroxisomes depends on MARCHF7 and PXMP4. Consistently, NBR1 strongly colocalized with peroxisomes in PEX1-depleted cells. However, when MARCHF7 or PXMP4 was additionally knocked down, the colocalization of peroxisomes with NBR1 was significantly reduced (Figure 6D,E), suggesting that MARCHF7-mediated PXMP4 ubiquitination is required for NBR1 recruitment to peroxisomes during PEX1 depletion-induced pexophagy.

**Figure 7.**
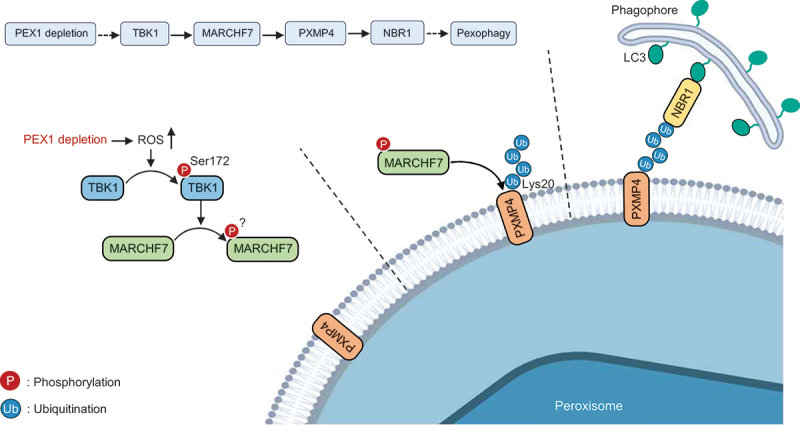
Schematic model of a novel regulatory axis mechanism for pexophagy. PEX1 depletion leads to peroxisomal dysfunction and an increase in reactive oxygen species (ROS) levels. Elevated ROS levels cause TBK1 phosphorylation at serine 172 (Ser172), leading to TBK1 activation. Activated TBK1 phosphorylates MARCHF7, which in turn promotes the ubiquitination of the peroxisomal membrane protein PXMP4 at lysine 20 (Lys20). The increased ubiquitination of PXMP4 facilitates the recruitment of the autophagy receptor protein NBR1, which mediates the degradation of dysfunctional peroxisomes through the autophagy pathway.

Taken together, these data support a regulatory model in which TBK1 activation, driven by PEX1 depletion-induced ROS, facilitates MARCHF7 phosphorylation, PXMP4 ubiquitination, and the subsequent recruitment of NBR1 to peroxisomes for degradation (Figure 7).

## Discussion

Peroxisomes are vital organelles that support diverse metabolic processes as well as redox homeostasis. Failure to regulate peroxisome dynamics and function can lead to various pathologies, including peroxisome biogenesis disorders and neurodegenerative diseases [3,66–71]. Indeed, degradation of peroxisomes by pexophagy has been implicated in conditions where peroxisome biogenesis is compromised [14,72,73]. However, the molecular signaling mechanisms that link peroxisomal stress to selective peroxisome turnover remain incompletely defined.

In this study, we identified a novel regulatory signaling scenario TBK1-MARCHF7-PXMP4-NBR1 axis that governs pexophagy in response to PEX1 depletion. Our findings emerged from a focused screen targeting members of the MARCHF E3 ubiquitin ligase family, which has been previously linked to various types of organelle-selective autophagy [53,74,75]. Among the screened candidates, MARCHF7 emerged as a previously unrecognized pivotal regulator of peroxisome turnover (Figure S2). This discovery underscores the functional diversity of MARCHF family E3 ubiquitin ligases in selective autophagy pathways, extending their known roles beyond mitochondria and the endoplasmic reticulum and into pexophagy.

In this study, we found that PEX1 depletion triggers peroxisomal dysfunction and a concomitant rise in ROS, ultimately culminating in pexophagy. We observed that treating PEX1-silenced cells with the ROS scavenger, NAC suppressed not only the phosphorylation TBK1 but also the ubiquitination of PXMP4 and the subsequent loss of peroxisomes (Figure 5). These data strongly indicates that ROS acts as a primary upstream signal to activate TBK1 when PEX1 function is compromised. Consistent with this notion, previous reports have also linked PEX1 depletion or mutation to higher ROS levels dysfunction [32,35,76], implying that this mechanism may be relevant in multiple models of peroxisome. While the precise molecular details of how ROS directly or indirectly leads to TBK1 activation remain to be fully clarified, our findings place ROS generation at the center of a signaling network that culminates in the selective degradation of damaged peroxisomes.

TBK1 is a serine/threonine kinase known for its role in innate immunity and selective autophagy, including mitophagy and lysophagy [64,65,77]. However, its involvement in pexophagy has remained largely unexplored. Our data further show that TBK1 becomes phosphorylated at Ser172 and subsequently phosphorylates MARCHF7, an E3 ubiquitin ligase required for peroxisome turnover under PEX1 depletion conditions (Figure 4). Importantly, the functional relevance of TBK1 phosphorylation is demonstrated by the finding that either chemical inhibitors or genetic depletion of TBK1 prevents the ubiquitination of peroxisomal proteins and the autophagic loss of peroxisomes (Figures 4E-J and 6C). Notably, MARCH family ligases can be phosphorylated by upstream kinases to modulate substrate selection and ubiquitination efficiency [78,79]. Our results indicate that TBK1 acts as an upstream regulator of MARCHF7 in pexophagy, laying the foundation for future studies to pinpoint the relevant phosphorylation sites on MARCHF7 and to detail how these post-translational modifications enhance its catalytic activity toward specific peroxisomal substrates.

Another central advance of our study is the identification of PXMP4 as a critical ubiquitination target in PEX1 depletion-induced pexophagy. While previous studies reported the ubiquitination of peroxisomal membrane proteins such as ABCD3 under oxidative stress or nutrient deprivation to promote peroxisome turnover [30,33,34], our findings expand the repertoire of peroxisomal membrane proteins that signal for pexophagy. Through a combination of computational prediction, site-directed mutagenesis, and ubiquitination assays, we determined that K20 of PXMP4 is the major site targeted by MARCHF7 (Figure 2). Significantly, MARCHF7-PXMP4 interaction and PXMP4 ubiquitination are enhanced specifically under PEX1 depletion (Figure 2E–G). Depleting *PXMP4*, in turn, blocks peroxisome loss, indicating that it serves as a key “eat-me” signal for the autophagic machinery during pexophagy (Figure 2A,B). Consistently, reconstitution of PXMP4 WT in PXMP4-depleted cells restored pexophagy, whereas the ubiquitination-defective PXMP4^K20R^ mutant failed to rescue pexophagy (Figure 3). On the other hand, a previous study reported that MARCHF7 mediates the ubiquitination of ATG14, thereby reducing the number of aggresome-like structures in osteosarcoma U2OS cells [80]. Therefore, further research is required to elucidate the regulatory mechanisms of ATG14 in pexophagy and to determine whether MARCHF7 ubiquitinates additional peroxisomal proteins. Additional exploration of the function of PXMP4—whether it has structural roles in the peroxisomal membrane or acts as a platform for docking specific regulatory factors – could further clarify how its ubiquitination triggers the recruitment of receptor proteins and cargo receptors.

A notable clinical implication of our study related with high frequency of PEX1 mutations in peroxisome biogenesis disorders, including Zellweger spectrum disorders, in which approximately 65% of cases are linked to PEX1 mutations [27]. Our current findings add to accumulating evidence that PEX1 dysfunction not only impairs normal peroxisome biogenesis but also drives excessive pexophagy via a TBK1-MARCHF7-PXMP4 axis. Notably, previous studies showed that chloroquine-mediated autophagy inhibition can help rescue peroxisome numbers and metabolic function in cells harboring PEX1 mutations [34]. This suggests that therapeutically modulating the newly identified TBK1-MARCHF7-PXMP4 axis could mitigate the pathological loss of peroxisomes in PEX1-associated disorders. Although caution is necessary when considering global suppression of autophagy – given its essential roles in many cellular housekeeping processes – targeting specific steps in the pathway, such as blocking TBK1 phosphorylation of MARCHF7 or inhibiting the MARCHF7-PXMP4 interaction, might permit a more tailored therapeutic intervention. Therefore, further investigations in patient-derived cells or animal models will be critical to evaluate the feasibility and safety of such strategies.

Collectively, our work reveals a central role for the ROS-TBK1-MARCHF7-PXMP4-NBR1 signaling cascade in orchestrating pexophagy under conditions of PEX1 depletion. By linking peroxisomal stress to a kinase-E3 ubiquitin ligase axis, we expand the emerging paradigm in which phosphorylation events fine-tune ubiquitin-mediated organelle degradation. This advancement opens new avenues for investigating how different kinases and E3 ubiquitin ligases integrate stress signals to maintain organelle homeostasis. Future research should explore how TBK1 discriminates among different organelle substrates in response to ROS, how the phosphorylation events of MARCHF7 precisely regulate its ligase activity, and whether other peroxisomal membrane proteins, in addition to PXMP4, serve as key ubiquitination targets. Furthermore, since another peroxisome stress sensor kinase, ATM, has been implicated in pexophagy under oxidative stress conditions [30], it would be informative to examine potential crosstalk or cooperation between ATM and TBK1 in PEX1 depletion models in the near future.

In summary, our findings provide a comprehensive molecular framework for how PEX1 depletion triggers a ROS-dependent pexophagy pathway. By unveiling TBK1 as an upstream kinase that phosphorylates MARCHF7, thereby promoting PXMP4 ubiquitination and recruitment of the NBR1 receptor, we establish a novel axis (TBK1-MARCHF7-PXMP4-NBR1) controlling peroxisomal clearance. Understanding these newly identified regulatory cascades not only offers insights into fundamental cell biology but also suggests therapeutic targets for peroxisome-related diseases.

## Materials and methods

### Reagents, siRNAs, and plasmids

Bafilomycin A_1_ (B1793) and N-acetyl-L-cysteine (NAC; A9165) were obtained from Sigma-Aldrich. GSK8612 (HY-111941) and MRT67307 (HY-13018) were purchased from MedChemExpress. MG132 (10012628) was obtained from Cayman Chemical Co. Validated short interfering RNAs (siRNAs) targeting *PEX1* (5’-GGACAAGAUUGGUGGGUUAUU-3’), *MARCHF7* (5’-GCACUUGGGAGUAAUUUGAUU-3”), *ABCD1* (5”-GCAUGAACCGGGUAUUCCUUU-3’), *ABCD2* (5’-GCAUGAUAAAGGUUAUACAUU-3’), *ABCD4* (5’-GCAUCAACACCUUUGACUAUU-3’), *SLC25A17* (5’-GGUCAAAGGUCAACAUUCUUU-3’), *PXMP4* (5′-CAGACUUCCUCGUCUAUAAUU-3′), *TBK1* (5′-GCAGUUUGUUUCUCUGUAU-3′), *NBR1* (5′-GAAGAGGUAUCCAUCAACAUU-3′), *SQSTM1/p62* (5′-GCAUUGAAGUUGAUAUCGAUUU-3′), and scrambled siRNA (5′-CCUACGCCACCAAUUUCGU-3′) were synthesized by Genolution. The HA-tagged pcDNA3.1 plasmid was obtained from Addgene (128034; deposited by Oskar Laur). The HA-tagged ubiquitin WT (HA-UB) plasmid was obtained from Addgene (17608; deposited by Ted Dawson). The pcDNA3.1+/C-PXMP4 WT-DYK plasmid was obtained from GenScript (OHu17287). mCherry-SEpHluorin-SKL was generated via subcloning into the pcDNA3 vector. The pEGFP-LC3 plasmid was a gift from Tamotsu Yoshimori (Osaka University, Japan).

### Mutagenesis

Site-directed mutagenesis was performed using the Muta-Directed Mutagenesis Kit (iNtRON Biotechnology, 15,071) to create PXMP4 K20R, K31R, and K207R mutants. The oligonucleotide primers for mutagenesis were PXMP4^K20R^ (Forward: 5′- AAC GCA CTG CTG CGC AGG CGC CGC TAC CAC GCT-3′ and Reverse: 5′-AGC GTG GTA GCG GCG CCT GCG CAG CAG TGC GTT-3′), PXMP4^K31R^ (Forward: 5′-GCT GCG TTG GCC GTG CTT AGG GGC TTC CGG AAC GGG GCT-3′ and Reverse: 5′-AGC CCC GTT CCG GAA GCC CCT AAG CAC GGC CAA CGC AGC-3′), and PXMP4^K207R^ (Forward: 5′-TTC CTC GTC TAT AAC AGG AGC CGT CCC TCC AAT-3′ and Reverse: 5′-ATT GGA GGG ACG GCT CCT GTT ATA GAC GAG GAA-3′). The oligonucleotide primers for si*PXMP4* resistant mutants were PXMP4 WT (Forward: 5′-GCA ATG TAT GGC ACG ACA TCT CAG ATT TTC TTG TTT ATA ACA AGA GCC GTC CCT CC-3′ and Reverse: 5′-GGA GGG ACG GCT CTT GTT ATA AAC AAG AAA ATC TGA GAT GTC GTG CCA TAC ATT GC-3′), and PXMP4^K20R^ (Forward: 5′-GCA ATG TAT GGC ACG AGG TCT CAG ATT TTC TTG TTT ATA ACA AGA GCC GTC CCT CC-3′ and Reverse: 5′-GGA GGG ACG GCT CTT GTT ATA AAC AAG AAA ATC TGA GAC CTC GTG CCA TAC ATT GC-3′). PXMP4 WT and PXMP4^K20R^ constructs resistant to si*PXMP4* were generated by site-directed mutagenesis (Cosmogenetech co. Ltd., Seoul, Korea). The specificity of the mutagenesis results was confirmed through DNA sequencing.

### Cell culture and establishment of stable Cell lines and siRNA screening

HeLa cells were obtained from the American Type Culture Collection (CCL-2). *ATG5*-KO HeLa cells, generated using the CRISPR-Cas9 system, were kindly provided by Dr. T. Kanki (Niigata University, Niigata, Japan) [81]. All cells were cultured at 37°C in a 5% CO_2_ incubator and maintained in DMEM (WELGENE, LM001-05) supplemented with 10% FBS (Hyclone, SH30919.03) and 1% penicillin-streptomycin (WELGENE, LS202-02).

For small-scale siRNA library screening, five peroxisomal membrane proteins were targeted. HeLa cells were seeded into 24-well plates, and after 24 h, each siRNA was transfected. Immunostaining was performed using an ABCD3 antibody (Abcam, ab3421) to label peroxisomes, and peroxisomal puncta were monitored using an I×71fluorescence microscope (Olympus, Tokyo, Japan).

### Western blotting and immunoprecipitation

Cell lysates were prepared using 2× Laemmli sample buffer (Bio-Rad, 1,610,737). Total protein concentration was measured using Bradford dye (Bio-Rad, 5,000,001), following the manufacturer’s instructions. Samples were separated by SDS-polyacrylamide gel electrophoresis (SDS-PAGE) and transferred onto a PVDF membrane (Bio-Rad, 1,620,177). After blocking with 4% skim milk (BD Bioscience, 90,002–594) in TBST (Tris base [GenDEPOT, T9200], Tween® 20 [Sigma, P7949], NaCl [GenDEPOT, G0610]), membranes were incubated with primary antibodies, including: anti-ABCD3 and anti-PEX1 (ab217059; 1:1000) antibodies were obtained from Abcam; anti-MARCHF7 (sc-166945; 1:1000), anti-p-Ser (sc-81514; 1:1000), and anti-HA (sc-7392; 1:1000) antibodies were purchased from Santa Cruz Biotechnology; anti-PXMP4 (NBP3-05927; 1:1000) antibody was purchased from NOVUS Biologicals; anti-DYKDDDDK (S2368; 1:1000), anti-p-Ser172-TBK1 (S5483; 1:1000), and anti-TBK1 (S3013; 1:1000) antibodies were purchased from Cell Signaling Technology; anti-ACTA1 (MAB1501; 1:10000) antibody was purchased from Sigma-Aldrich.

For detection, membranes were incubated with HRP-conjugated secondary antibodies (Cell Signaling Technology, 7076S, 7074S; 1:5000)

Cells were lysed for 1 h at 4°C in RIPA buffer (iNtRON Biotechnology, IBS-BR002) with protease inhibitors (GenDEPOT, P3100). The supernatant was incubated with antibodies against MARCHF7, TBK1, PXMP4, and FLAG-agarose for 24 h at 4°C. Protein A/G PLUS-agarose (Santa Cruz Biotechnology, sc-2003) was then added, and samples were incubated for 2–4 h at 4°C. Immunoprecipitated proteins were analyzed by western blotting.

### Confocal microscopy and determination of pexophagic cells

To quantify pexophagy, HeLa cells were cultured on glass coverslips, treated with chemicals, or transfected with siRNA for various durations. The cells were then washed with PBS (WELGENE, LB 001–02), fixed with 4% paraformaldehyde (Biosesang, P2031) for 20 min, and stained with the indicated dyes or antibodies. Punctate peroxisomal structures were visualized using a confocal microscope (Carl Zeiss, LSM 800; Leica, STELLARIS 5). The number of peroxisome puncta was quantified with ABCD3 protein using ImageJ Fiji. Experiments were conducted at least three times for each condition, as indicated in each figure. The peroxisome count was determined by dividing the number of peroxisomal structures by the cell volume.

### Measurement of ROS levels

Cellular ROS levels were evaluated using the fluorescent dye 2“,7”-dichlorodihydrofluorescein diacetate (DCFH-DA; Thermo Fisher Scientific, C2938). HeLa cells were transiently transfected with siRNA targeting *PEX1*, with or without NAC treatment. After 48 h, cells were incubated with DCFH-DA for 10 min, and fluorescence was measured using ImageJ software. Each experiment was conducted at least three times for each condition. Fluorescence intensity was determined by analyzing 300 cells, based on measurement time points.

### Colocalization assay

For colocalization analysis, HeLa cells were transfected with the indicated siRNAs or DNA constructs and cultured for 48 h or 72 h prior to imaging, then washed, fixed, and immunostained with antibodies against ABCD3, MARCHF7, Flag (Sigma-Aldrich, F1804; 1:1000), and NBR1 (Santa Cruz Biotechnology, sc-130380; 1:1000). Confocal microscopy (Zeiss LSM 800, Carl Zeiss; STELLARIS 5, Leica Microsystems) was used for imaging. Colocalization was quantified by calculating Pearson’s correlation coefficient with the Coloc2 plugin in ImageJ Fiji, and results were presented graphically.

### Statistical analyses

Colocalization analysis was performed using ImageJ Fiji and Pearson’s correlation coefficient. Data were collected from at least three independent experiments and presented as mean ± standard error of the mean (SEM). Statistical analysis was conducted using one-way ANOVA, with *p* < 0.05 considered significant.

## Supplementary Material

MARCHF7_Supplementary_data_manuscript_20251030 R3.docx

## Data Availability

Data supporting the findings of this study are available from the corresponding author upon reasonable request.

## Abbreviations

Baf.A1: bafilomycin A_1_
DCFH-DA: 2“,7”-dichlorodihydrofluorescein diacetate
ERphagy: endoplasmic reticulum (ER) selective autophagy
H_2_O_2_: hydrogen peroxide
KO: knockout
NAC: N-acetyl-L-cysteine
ROS: reactive oxygen species
PEX: peroxisomal biogenesis factors
PTS: peroxisomal-targeting signal
VLCFAs: very-long-chain fatty acids.
