# Supplementary material for: Regulation of pexophagy by a novel TBK1-MARCHF7-PXMP4-NBR1 axis in PEX1-depleted HeLa cells
Source: Autophagy. 2025 Nov 27;22(2):316–29. doi: 10.1080/15548627.2025.2593585 (PMC12834161; doi:10.1080/15548627.2025.2593585)
Supplement: MARCHF7_Supplementary_data_manuscript_20251030 R3.docx [file KAUP_A_2593585_SM1981.docx]

**Supplementary Data**

**Regulation of Pexophagy by a Novel TBK1-MARCHF7-PXMP4-NBR1 Axis in PEX1-Depleted HeLa Cells**

Yong Hwan Kim^a^, Joon Bum Kim^b^, Ji-Eun Bae^b^, Na Yeon Park^b^, Seong Hyun Kim^a^, Jae-Young Um^c^, Dong-Seok Lee^a^, Kyu Sun Lee^d^, Peter K. Kim^e^, Doo Sin Jo^f*^, and Dong-Hyung Cho^a,b,^^f*^

^a^School of Life Sciences, BK21 FOUR KNU Creative BioResearch Group, Kyungpook National University, Daegu 41566, Republic of Korea; ^b^Organelle Institute, Kyungpook National University, Daegu 41566, Republic of Korea; ^c^Department of Science in Korean Medicine, Graduate School, Kyung Hee University, 02447, Seoul, Republic of Korea; ^d^Metabolism and Neurophysiology Research Group, KRIBB, Daejeon, Republic of Korea; ^e^Department of Biochemistry, University of Toronto, Toronto, ON, M5S 1A8, Canada; ^f^ORGASIS Corp. Suwon, Gyeonggi-do, 16229, Republic of Korea

^*^Corresponding Author:

Doo Sin Jo Ph.D.; ORGASIS Corp. Gyeonggido Business & Science Accelerator, Suwon, Gyeonggi-do, 16229, Republic of Korea

Tel: 82-31-211-0316, Email: doosinjo@gmail.com

Dong-Hyung Cho Ph.D.; School of Life Sciences, Kyungpook National University, 80 Daehakro Bukgu, Daegu 41566, Republic of Korea

Tel: 82-53-950-5382; E-mail: [dhcho@knu.ac.kr](mailto:dhcho@knu.ac.kr) (D.-H.C)

**Supplementary Materials and Methods**

***Reagents, siRNAs and plasmids***

Short interfering RNA (siRNA) targeting *MARCHF7* (#2, 5’-GCACACGUGUCCGAUUUAUUU-3') and *TBK1* (#1, 5’-CCAUGUGGGAGUUUAUACA-3’) were synthesized by Genolution.

***Cell culture and establishment of stable cell lines and siRNA screening***

For small-scale siRNA library screening, a small-scale siRNA library targeting 11 members of the *MARCHF* E3 ubiquitin ligase family was constructed. HeLa cells were seeded into 24-well plates, and after 24 h, each siRNA was transfected. Immunostaining was performed using an ABCD3 antibody to label peroxisomes, and peroxisome puncta were monitored using confocal microscope (Carl Zeiss, LSM 800).

***PPARA, PPARD and PPARG activity assay***

PPARA, PPARD, and PPARG were detected with isoform-specific primary antibodies (Abcam, ab133113). HeLa cells were transfected with scrambled siRNA or *PEX1*-targeting siRNA, and PPAR activity was determined with the PPAR (A, D, G) Transcription Factor Assay Kit (Abcam, ab133113). HRP-conjugated secondary antibodies were applied, and absorbance was measured at 450 nm using a microplate reader (BioTek, SYNERGY H1).

***Quantification and statistical analysis***

Data were obtained from at least three independent experiments and were presented as mean ± standard error of the mean. Statistical evaluation of the results was performed using one-way analysis of variance. p-values of 0.05 were considered to indicate statistical not significance.

**Table S1.** *MARCHF* E3 ubiquitin ligase family-targeted small-scale siRNA library list.

| siRNA | Sequence |
| --- | --- |
| si*MARCHF1* | 5’-CAGGAGGUCUUGUCUUCAUUU-3’ |
| si*MARCHF2* | 5’- CCGUGCAUAAGAGCUGUCUGGAGAAUU-3’ |
| si*MARCHF3* | 5’- UGGAGGAUUGUGGCAGCCUAGUGAAUU-3’ |
| si*MARCHF4* | 5’- CAGAUCUGCUACGGGAUGUUU-3’ |
| si*MARCHF5* | 5’- GGACAGCUGUGACUUAUGGUU-3’ |
| si*MARCHF6* | 5’-CACCAAAGCUGUUGUACAUUU-3’ |
| si*MARCHF7* | 5’-GCACUUGGGAGUAAUUUGAUU-3’ |
| si*MARCHF8* | 5’-CUCCAAGCCAGUGUCUGAUUU-3’ |
| si*MARCHF9* | 5’- GCAGUGGAAGGUCCUAAAUUAUU-3’ |
| si*MARCHF10* | 5’- CGUCACAGCCAAUGACUGAUU-3’ |
| si*MARCHF11* | 5’-GUCUUCCACAAGUAGGACU-3’ |

A small-scale siRNA library targeting 11 members of the *MARCHF* E3 ubiquitin ligase family was constructed.


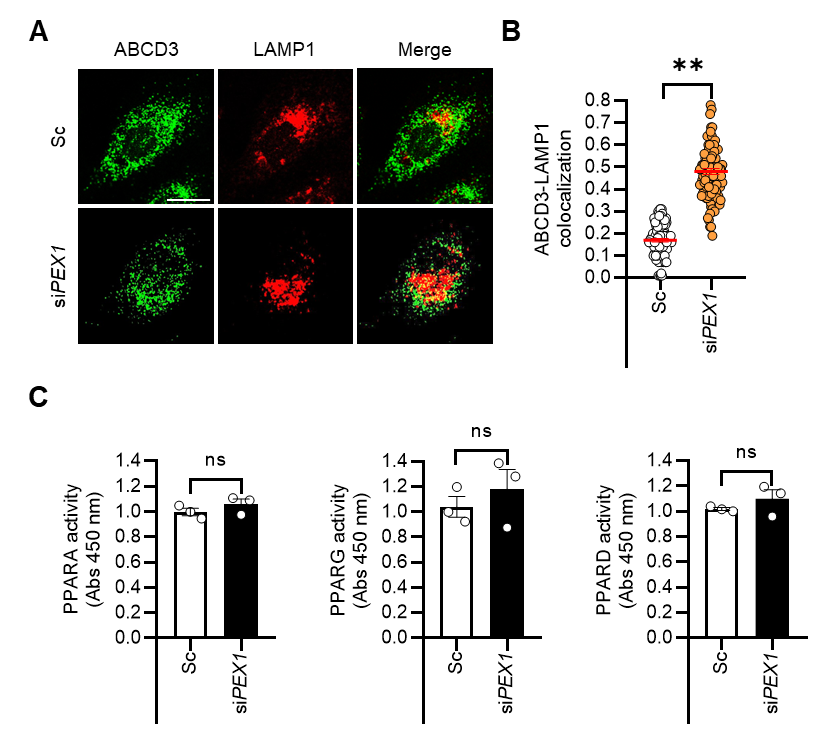


**Figure S1.** Depletion of PEX1 induces peroxisome loss in an autophagy-dependent manner. (**A and B**) HeLa cells were transfected with scrambled siRNA (Sc) or si*PEX1* for 72 h. Cells were then fixed, immunostained with anti-ABCD3 and anti-LAMP1 antibodies, and imaged using confocal microscopy. Pearson’s correlation coefficient was used to quantify the colocalization between ABCD3 and LAMP1. The graph bars represent the colocalization analysis results obtained using the Coloc2 plugin in Fiji, based on Pearson’s correlation coefficient (*n* ≥ 100). (**C**) HeLa cells were transfected with Sc or si*PEX1* for 72 h, and PPAR activity was determined using the PPAR (A, D, G) Transcription Factor Assay Kit (*n* = 3). Scale bar: 20 µm. Data are presented as mean ± SEM; ***p* < 0.001; ns, not significant.

**Figure S2.** Identification of a novel E3 ligase involved in pexophagy. HeLa cells were transiently transfected with Sc or si*PEX1* in combinations with a *MARCHF* E3 ubiquitin ligase family siRNAs for 72 h. Then, the cells were stained with anti-ABCD3 antibody. The number of peroxisomal puncta per cell was calculated (*n* ≥ 100). Data are presented as mean ± standard error of the mean; ** *p* < 0.001.

**
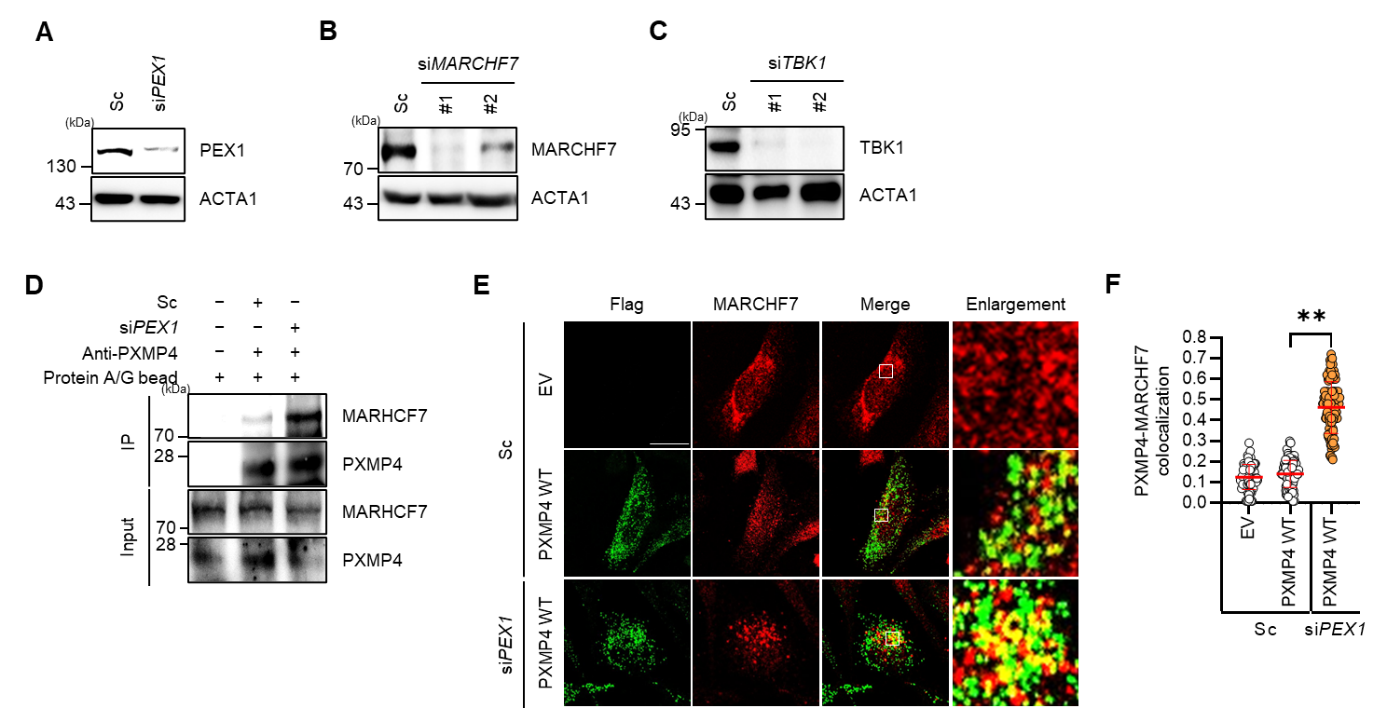
**

**Figure S3.** MARCHF7 interacts with PXMP4. (**A**) HeLa cells were transiently transfected with siRNA targeting *PEX1* (si*PEX1*) for 72 h, then harvested for analysis by Western blotting using the indicated antibodies. The reduced expression of PEX1 by siRNA was validated. (**B**) HeLa cells were transiently transfected with siRNA targeting *MARCHF7* (si*MARCHF7 #1* and *#2*) for 72 h, then harvested for analysis by western blotting using the indicated antibodies. The reduced expression of MARCHF7 by siRNA was validated. (**C**) HeLa cells were transiently transfected with siRNA targeting *TBK1* (si*TBK1 #1* and *#2*) for 72 h, then harvested for analysis by western blotting using the indicated antibodies. The reduced expression of MARCHF7 by siRNA was validated. (**D**) HeLa cells were transiently transfected with scrambled siRNA (Sc) or *PEX1* siRNA (si*PEX1*) for 72 h. After, the cells were harvested and subjected to immunoprecipitation by using anti-PXMP4 antibodies conjugated to agarose beads. The samples were analyzed by western blotting by using the indicated antibodies. (**E and F**) HeLa cells were co-transfected with either Flag (Empty vector, EV) or PXMP4 WT-Flag (PXMP4 WT) vectors together with Sc or si*PEX1* for 48 h. Cells were then fixed, immunostained with anti-MARCHF7 and anti-Flag antibodies, and imaged using confocal microscopy. Colocalization between Flag (PXMP4) and MARCHF7 was quantified using Pearson’s correlation coefficient. Graph represents values obtained with the Coloc2 plugin in Fiji (*n* ≥ 100). Scale bar: 20 µm. Data are presented as mean ± standard error of the mean (SEM); ***p* < 0.001.
